# Exploring angiogenic pathways in breast cancer: Clinicopathologic correlations and prognostic implications based on gene expression profiles from a large-scale genomic dataset

**DOI:** 10.1371/journal.pone.0310557

**Published:** 2024-09-20

**Authors:** Nehad M. Ayoub, Salam Sardiah, Qusai Y. Al-Share, Mohammad S. Alkader

**Affiliations:** 1 Department of Clinical Pharmacy, Faculty of Pharmacy, Jordan University of Science and Technology, Irbid, Jordan; 2 Department of Medical Oncology, Military Cancer Center, Jordanian Royal Medical Services, Amman, Jordan; Universita degli Studi della Campania Luigi Vanvitelli, ITALY

## Abstract

**Background:**

Angiogenesis inhibitors targeting VEGF, or its receptors have consistently produced disappointing clinical outcomes in breast cancer. Therefore, there is an urgent need to explore alternative angiogenic pathways in breast cancer. This study aimed to describe the gene expression of pivotal pro-angiogenic genes in breast cancer and to further analyze the associations with the clinicopathologic tumor features, prognostic factors, and overall survival. Such findings would expand the understanding of the role of different angiogenic pathways in breast cancer pathogenesis and identify patients at risk of more aggressive disease who could be eligible for intense treatment regimens. Additionally, exploring angiogenic pathways helps identify new potential drug targets for breast cancer.

**Methods:**

The mRNA expression levels for eight pro-angiogenic genes [*VEGFA*, *HGF*, *FGF1*, *FGF2*, *ANGPT1*, *ANGPT2*, *PDGFA*, *and PDGFB*] were obtained from the METABRIC (Molecular Taxonomy of Breast Cancer International Consortium) dataset available at cBioPortal public domain. Pertinent demographic and tumor information were retrieved.

**Results:**

VEGFA and ANGPT2 genes had the highest expression levels with average mRNA log intensities of 7.18±0.7 and 7.11±0.53, respectively. *VEGFA* expression was not correlated with the expression of other pro-angiogenic genes, the clinicopathologic tumor features, and the overall survival of patients. *FGF1*, *ANGPT1*, and *PDGFA* mRNA levels were negatively correlated with the age of patients at diagnosis. The expression of *FGF1* and *FGF2* correlated inversely with tumor size and the Nottingham Prognostic Index (p = 0.03 and p = 0.002, respectively). Expression of *HGF* was significantly associated with advanced tumor stage (p<0.05). Expression of *ANGPT1* and *ANGPT2* was associated with hormone receptor-negative status and the non-luminal subtypes. *PDGFB* expression was significantly higher in patients with high-grade disease and HER2-positive status. Patients with high expression status of *ANGPT2* and *PDGFB* had significantly reduced overall survival compared to those with low expression levels of these genes (p = 0.004 and p = 0.0001, respectively).

**Conclusions:**

In this dataset of patients with breast cancer, the expression levels of 8 different pro-angiogenic genes revealed remarkable differences in terms of their association with clinicopathologic tumor characteristics and prognosis. The expression of *ANGPTs* and *PDGFs* was associated with adverse tumor features, worse prognosis, and reduced survival in patients. Targeting ANGPTs and PDGF pathways could provide new insights for effective anti-angiogenic drugs in breast cancer.

## Introduction

Angiogenesis is the formation of new blood vessels from the existing vasculature [1]. It is an essential process for various physiological conditions such as embryonic development, tissue repair, wound healing, muscle growth, and organ lining regeneration [2, 3]. Angiogenesis is controlled by a complex network of signaling pathways involving various growth factors, cytokines, and enzymes. Under normal physiological conditions, endothelial cells exist in a quiescent non-proliferative state [2, 4]. However, biological signals in response to vascular injury, hypoxia, and/or inflammation upregulate the expression of pro-angiogenic growth factors to activate quiescent endothelial cells and stimulate vascular growth [4]. Angiogenic growth consists of several highly regulated sequential steps that include the degradation of the basement membrane, the proliferation and migration of the endothelial cells, and the stabilization and maturation of the newly formed blood vessels [2–4]. Angiogenesis is tightly controlled by the balance between a variety of pro-angiogenic and anti-angiogenic factors [4]. Under specific pathological conditions, this regulation is disturbed as a result of the overexpression of pro-angiogenic factors and/or the inactivation of anti-angiogenic factors leading to excessive vascular growth [2–4]. Pathologic angiogenesis is a key feature in a variety of conditions such as rheumatoid arthritis, psoriasis, diabetic retinopathy, age-related macular degeneration, atherosclerosis, and cancer [2, 3]. Angiogenesis plays a critical role in the growth and progression of solid cancers. It provides the tumor mass with the vascular networks essential to supply oxygen and nutrients for tumor growth, invasion, and metastasis [5, 6]. For the growth and progression of solid cancers, the normal balance between pro-angiogenic and anti-angiogenic factors is altered in favor of increased secretion of pro-angiogenic factors and is described as the ‘*angiogenic switch’* [7]. The angiogenic switch is regularly activated by an increased growth rate of the cancer cells and the hypoxic microenvironment which together induce the secretion of multiple pro-angiogenic growth factors by tumor cells into the surrounding microenvironment [6, 8]. Unlike normal blood vessels, the newly formed tumor vasculature is characterized by several functional and structural abnormalities such as leakiness and tortuosity [9]. These alterations lead to abnormal blood flow resulting in reduced delivery and perfusion of chemotherapeutic drugs to tumor tissue [5, 6]. Several pro-angiogenic factors regulate tumoral vascular growth such as the vascular endothelial growth factor (VEGF), the fibroblast growth factors (FGFs), angiopoietins (ANGPTs), and platelet-derived growth factors (PDGFs) [10, 11]. Among these, VEGF is a crucial pro-angiogenic factor and a key regulator of angiogenesis. By binding to its target receptors on endothelial cells, VEGF mediates the remodeling of the extracellular matrix, increases vascular permeability, and maintains the survival of the newly formed blood vessels during angiogenic growth [12, 13]. The VEGF family includes VEGF-A, B, C, D, E, and the placental growth factor. These factors produce their effects through the activation of VEGF receptors (VEGFRs) which belong to class III receptor tyrosine kinase (RTKs): VEGFR-1, VEGFR-2, and VEGFR-3 [14, 15]. VEGF-A is the most potent among the VEGF family and it binds both VEGFR-1 and 2 [16]. Apart from its vascular effects, the activation of the VEGF/VEGFR signaling has been shown to suppress the antitumor immune cell response in the tumor microenvironment further promoting cancer cell survival and proliferation [15, 17]. Accordingly, angiogenesis inhibitors that target VEGF and/or its receptors are currently available for clinical use as anticancer drugs. These include monoclonal antibodies and small-molecule tyrosine kinase inhibitors (TKIs) that are frequently used for the treatment of several types of solid tumors including colorectal, kidney, ovarian, and lung cancers [18]. Despite the well-established efficacy of anti-angiogenic drugs in the treatment of advanced solid cancers, inhibitors of the VEGF/VEGFR pathway have consistently produced disappointing clinical outcomes in certain types of tumors, including breast cancer [4, 19].

Breast cancer is the most frequently diagnosed cancer and the second leading cause of cancer deaths in women worldwide [20]. It accounted for 2.3 million newly diagnosed cases and 685,000 deaths globally in 2020 [21]. Breast cancer is a heterogeneous disease that is composed of different molecular subtypes based on gene expression profiling. These subtypes include luminal A, luminal B, human epidermal growth factor receptor 2 (HER2)-positive, normal-like, and basal-like cancers [22, 23]. The subtypes differ in terms of disease prognosis, clinical outcomes, and the therapeutic targets they express [24]. Previous studies revealed that VEGF expression is abundant in breast cancer. VEGF was a prominent angiogenic factor in breast cancer biopsies from patients with the early stages of the disease [25, 26]. Furthermore, the aggressiveness and likelihood of invasive breast cancer correlated with higher levels of VEGF [19, 27, 28]. Higher VEGF expression in breast tumors was associated with advanced prognosticators including larger tumor size, high histologic grade, hormone receptor-negative status, HER2-overexpression, and lymph node metastasis [29–32]. Despite the high expression of VEGF in breast tumors and its adverse impact on the clinicopathologic features, angiogenesis inhibitors targeting VEGF and/or its receptors have consistently failed to produce favorable treatment outcomes in breast cancer [4]. In this context, several inhibitors of the VEGF/VEGFR pathway such as the monoclonal antibodies bevacizumab and ramucirumab, as well as the TKIs sorafenib, sunitinib, vandetanib, axitinib, pazopanib, and cediranib, did not provide survival advantage in patients with breast cancer in clinical trials [4]. Therefore, the angiogenic role of the VEGF/VEGFR pathway in breast cancer has been brought into question and such findings call for exploring alternative molecular pathways that could play a role in breast cancer angiogenesis. Such analysis would also provide insights into novel drug targets to regulate tumor angiogenesis that could be further investigated in clinical settings. Accordingly, the goal of this study was to assess the expression profile of a panel of genes encoding for pro-angiogenic growth factors in patients with breast cancer and to describe its relationship with clinicopathologic tumor characteristics and overall survival (OS). Findings from this study could reveal the molecular mechanisms of angiogenic growth in breast cancer and identify patients who are at higher risk of worse prognosis and could be eligible to more aggressive therapy.

## Methods

### The METABRIC dataset

The METABRIC (Molecular Taxonomy of Breast Cancer International Consortium) dataset provides genomic and transcriptomic data for more than 2000 primary breast tumors [33]. The breast tumors in the METABRIC cohort were collected from five centers in the United Kingdom and Canada [34]. The dataset is made freely available through the cBioPortal open-access public domain for cancer genomic datasets [https://www.cbioportal.org] [35]. The dataset was downloaded and the demographic, clinical, and tumor data of patients were retrieved. Such data include age at diagnosis, menopausal status, the Nottingham Prognostic Index (NPI), OS, the expression status of hormone receptors and HER2, the molecular subtypes, tumor stage, grade, number of positive lymph nodes, the tumor size, and the treatment received. Besides, the METABRIC dataset provides gene expression data as well as the putative gene copy number alterations for hundreds of genes. For this study, the mRNA gene expression for the pro-angiogenic genes *VEGFA*, *HGF*, *FGF1*, *FGF2*, *ANGPT1*, *ANGPT2*, *PDGFA*, and *PDGFB* were available for 1904 patients in the dataset for which they were retrieved and analyzed.

### Statistical analysis

The SPSS statistical package, version 23.0 (IBM Corp, Armonk, NY) was used for data analysis. Continuous variables are expressed as the mean ± standard deviation and categorical variables are expressed as frequencies and percentages. The means of two independent groups were compared using the independent student t-test. To assess correlations between continuous variables, Pearson’s correlation was applied. The dichotomization of some categorical variables was considered before conducting statistical analysis to avoid a small sample size upon further data stratification as previously described [36]. For survival analysis, the patients were stratified into *low-* and *high*-gene expression groups according to the mean log intensity value for each gene of interest. The low-expression group had an mRNA log intensity less than or equal to the mean while the high-expression group had an mRNA log intensity above the mean value. Kaplan-Meier survival curves were generated for patients according to the gene expression status using GraphPad Prism, version 8.0.1, software (GraphPad Software, San Diego, CA). All p values were two-sided, and the findings were statistically significant at p<0.05.

### Ethical approval

This study was approved by the Institutional Review Board of Jordan University of Science and Technology (Research No. 50/151/2022).

## Results

### Description of patients in the METABRIC dataset

A summary of the demographic and clinicopathologic characteristics of breast cancer patients from the METABRIC dataset has been previously published [36].

### Expression of Pro-angiogenic genes in patients with breast cancer

A total of 1904 samples of breast carcinoma were analyzed for 8 pro-angiogenic genes at the mRNA level. The average mRNA expression log intensity was the highest for *VEGFA* (7.18±0.7, range: 5.82–10.8) followed by *ANGPT2* (7.11±0.53, range: 5.68–9.33), and *PDGFB* (7.06±0.54, range: 5.64–9.47) compared to other genes as shown in Table 1. *HGF* was least expressed in patients with an average mRNA expression log intensity of 5.42±0.18 (range: 4.87–6.24).

**Table 1:** mRNA expression of pro-angiogenic genes in patients with breast cancer.

Characteristic	Pro-angiogenic Gene							
	*VEGFA*	*HGF*	*FGF1*	*FGF2*	*ANGPT1*	*ANGPT2*	*PDGFA*	*PDGFB*
**mRNA expression, log intensity**								
Mean ± SD	7.18±0.7	5.42±0.18	5.67±0.26	5.88±0.43	6.10±0.52	7.11±0.53	6.06±0.34	7.06±0.54
Range	5.82–10.8	4.87–6.24	5.10–7.21	4.95–9.53	5.09–9.88	5.68–9.33	5.11–8.09	5.64–9.47
**mRNA expression status, n (%)**								
Low	1102 (57.9)	983 (51.6)	1062 (55.8)	1206 (63.3)	1183 (62.1)	998 (52.4)	1064 (55.9)	1086 (57.0)
High	802 (42.1)	921 (48.4)	842 (44.2)	698 (36.7)	721 (37.9)	906 (47.6)	840 (44.1)	818 (43.0)

n (%), frequency and valid percentage.

ANGPT1, angiopoietin 1; ANGPT2, angiopoietin 2; FGF1, fibroblast growth factor 1; FGF2, fibroblast growth factor 2; HGF, hepatocyte growth factor; PDGFA, platelet-derived growth factor A; PDGFB, platelet-derived growth factor B; SD, standard deviation; VEGFA, vascular endothelial growth factor A.

Table 2 shows the correlation analysis for the panel of pro-angiogenic genes examined in this study. The mRNA expression level of *VEGFA* was not significantly correlated with the expression of any other gene. Interestingly, the mRNA expression level of *HGF* was inversely correlated with each of *ANGPT2* and *PDGFA* mRNA levels. Both *FGF1* and *FGF2* mRNA levels were positively correlated with *ANGPT1* and *PDGFA* (p<0.01), while inversely correlated with the expression of *PDGFB* (p<0.01). Besides, the expression of *ANGPT1* and *ANGPT2* as well as *PDGFA* and *PDGFB* correlated positively (p<0.01).

**Table 2:** Correlation analysis of mRNA expression of pro-angiogenic genes in patients with breast cancer.

Pro-angiogenic Gene	Pro-angiogenic Gene							
	*VEGFA*	*HGF*	*FGF1*	*FGF2*	*ANGPT1*	*ANGPT2*	*PDGFA*	*PDGFB*
***VEGFA***								
r	-------	0.019	0.020	– 0.020	0.005	– 0.014	0.024	– 0.009
***HGF***								
r	0.019	-------	0.002	0.027	– 0.017	– 0.049*	– 0.060**	– 0.039
***FGF1***								
r	0.020	0.002	-------	0.034	0.164**	0.170**	0.095**	– 0.086**
***FGF2***								
r	– 0.020	0.027	0.034	-------	0.186**	0.013	0.075**	– 0.075**
***ANGPT1***								
r	0.005	– 0.017	0.164**	0.186**	-------	0.165**	0.107**	– 0.037
***ANGPT2***								
r	– 0.014	– 0.049*	0.170**	0.013	0.165**	-------	0.155**	0.117**
***PDGFA***								
r	0.024	– 0.060**	0.095**	0.075**	0.107**	0.155**	-------	0.153**
***PDGFB***								
r	– 0.009	– 0.039	– 0.086**	– 0.075**	– 0.037	0.117**	0.153**	-------

mRNA expression in log intensity. r, Pearson’s correlation coefficient.

*p<0.05

**p<0.01.

ANGPT1, angiopoietin 1; ANGPT2, angiopoietin 2; FGF1, fibroblast growth factor 1; FGF2, fibroblast growth factor 2; HGF, hepatocyte growth factor; PDGFA, platelet-derived growth factor A; PDGFB, platelet-derived growth factor B; VEGFA, vascular endothelial growth factor A.

### The association of the expression of pro-angiogenic genes with clinicopathological characteristics of patients with breast cancer

The correlation analysis revealed that *FGF1*, *ANGPT1*, and *PDGFA* mRNA levels were negatively correlated with the age of the patient at diagnosis (Table 3). Alternatively, the mRNA levels of *FGF2* correlated positively with the age of patients (r = 0.083, p<0.001). Solely, *FGF1* expression was inversely correlated with tumor size (r = – 0.05, p = 0.03). *PDGFA* gene expression negatively correlated with the number of lymph nodes and NPI (Table 3). Alternatively, NPI was positively correlated with *ANGPT1* mRNA levels expressed in breast tumor tissues (r = 0.079, p<0.001). Based on the values generated for the coefficients in the bivariate correlation analysis, these correlations were considered weak in magnitude despite the significant findings. *VEGFA*, *HGF*, *ANGPT2*, and *PDGFB* mRNA levels were not correlated with any of the clinicopathologic features described in this analysis (Table 3).

**Table 3:** Correlation analysis of mRNA expression of pro-angiogenic genes with clinicopathologic characteristics of patients with breast cancer.

Characteristic	Pro-angiogenic Gene							
	*VEGFA*	*HGF*	*FGF1*	*FGF2*	*ANGPT1*	*ANGPT2*	*PDGFA*	*PDGFB*
**Age, years**								
r	– 0.022	0.031	– 0.180***	0.083***	– 0.129***	– 0.038	– 0.048*	– 0.007
**Tumor size, mm**								
r	0.002	0.002	– 0.050*	– 0.006	0.002	0.030	0.003	0.014
**Positive lymph nodes**								
r	0.013	0.013	– 0.027	– 0.032	0.012	0.018	– 0.054*	0.038
**Nottingham Prognostic Index**								
r	0.009	0.044	– 0.037	– 0.073**	0.079**	0.028	– 0.078**	0.037

mRNA expression in log intensity. r, Pearson’s correlation coefficient.

*p<0.05

**p<0.01

***p<0.001.

ANGPT1, angiopoietin 1; ANGPT2, angiopoietin 2; FGF1, fibroblast growth factor 1; FGF2, fibroblast growth factor 2; HGF, hepatocyte growth factor; PDGFA, platelet-derived growth factor A; PDGFB, platelet-derived growth factor B; VEGFA, vascular endothelial growth factor A.

**Fig 1:**
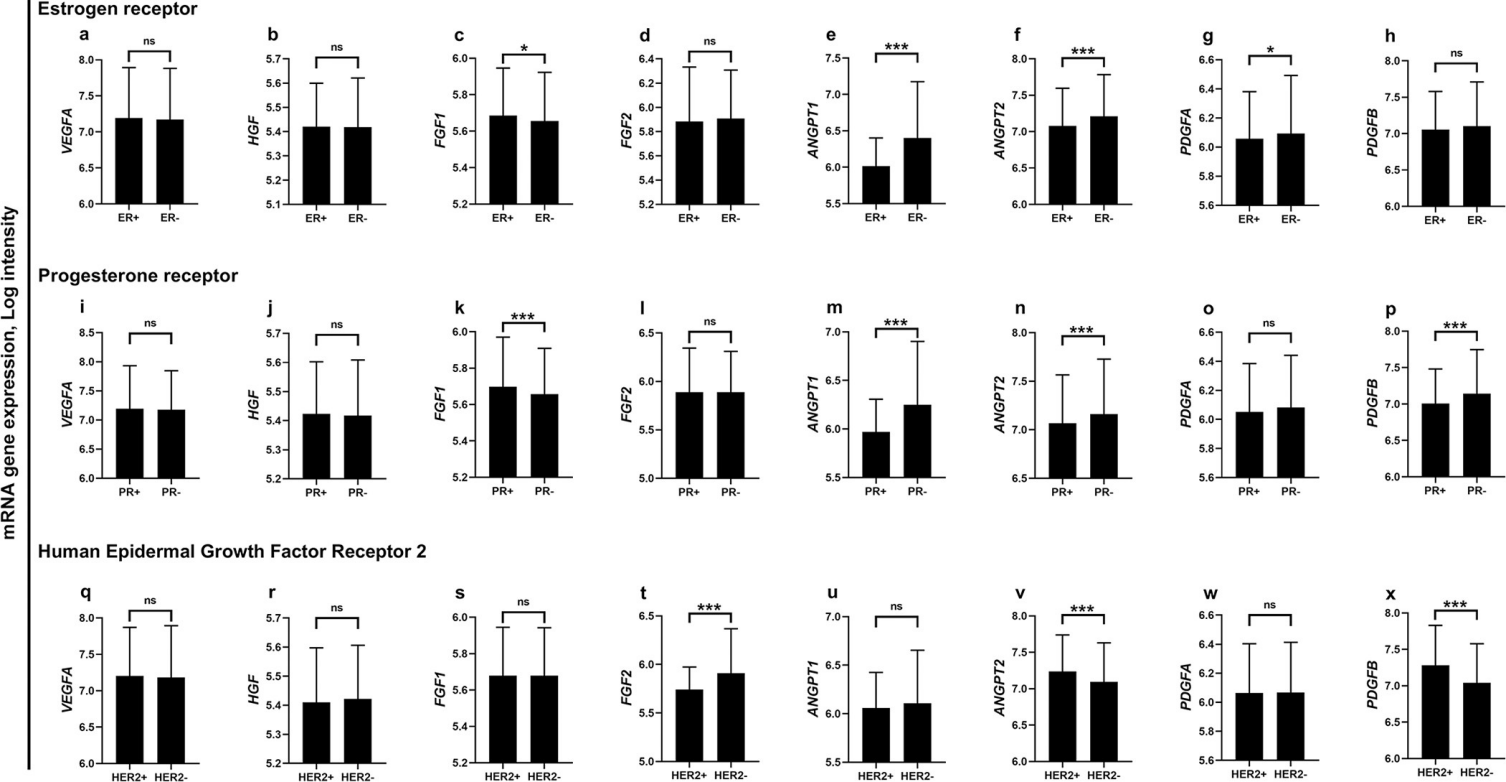
Expression of pro-angiogenic genes based on receptor status in patients with breast cancer. The mRNA expression levels of pro-angiogenic genes according to the expression status of ER (a-h), expression status of PR (i-p), and expression status of HER2 receptor (q-x). *p<0.05, ***p<0.001. ns, no statistically significant difference. Bars represent mean mRNA gene expression log intensity ± Standard deviation. ANGPT1, angiopoietin 1; ANGPT2, angiopoietin 2; ER, estrogen receptor; FGF1, fibroblast growth factor 1; FGF2, fibroblast growth factor 2; HER2, human epidermal growth factor receptor 2; HGF, hepatocyte growth factor; PDGFA, platelet-derived growth factor A; PDGFB, platelet-derived growth factor B; PR, progesterone receptor; VEGFA, vascular endothelial growth factor A.

The mean mRNA expression levels of *VEGFA* and *HGF* were not significantly different according to receptor status (Fig 1). The mean mRNA log intensity of *FGF1* was significantly higher in patients harboring hormone receptor-positive tumors compared with hormone receptor-negative ones (Fig 1C and 1K). Alternatively, the mean mRNA expression levels of *ANGPT1* and *ANGPT2* were significantly higher in patients with hormone receptor-negative status compared to patients with hormone receptor-positive tumors (p<0.001, Fig 1E, 1F, 1M and 1N). A similar pattern was also observed for the mRNA expression of *PDGFA* and *PDGFB* levels according to hormone receptor status (Fig 1). Patients with HER2-positivity had significantly higher mRNA levels of *ANGPT2* and *PDGFB* compared to those with HER2-negative status (p<0.001, Fig 1V and 1X).

**Fig 2:**
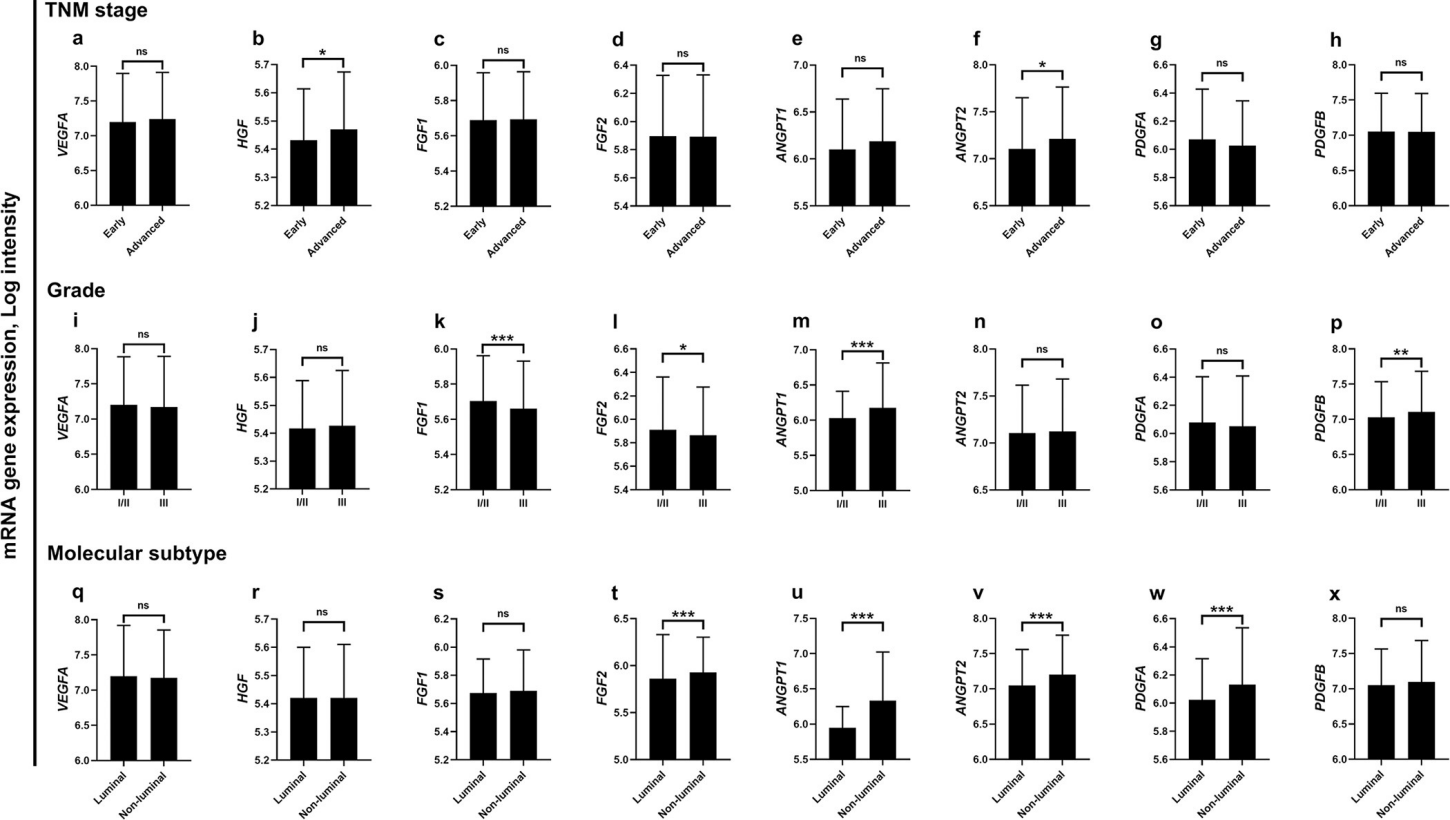
Expression of pro-angiogenic genes based on stage, grade, and molecular subtype in patients with breast cancer. The mRNA expression levels of pro-angiogenic genes according to tumor TNM stage (a-h), grade (i-p), and molecular subtype (q-x). *p<0.05, **p<0.01, ***p<0.001. ns, no statistically significant difference. Bars represent mean mRNA gene expression log intensity ± Standard deviation. ANGPT1, angiopoietin 1; ANGPT2, angiopoietin 2; FGF1, fibroblast growth factor 1; FGF2, fibroblast growth factor 2; HGF, hepatocyte growth factor; PDGFA, platelet-derived growth factor A; PDGFB, platelet-derived growth factor B; VEGFA, vascular endothelial growth factor A.

Fig 2 shows the mRNA expression levels of the pro-angiogenic genes based on tumor stage, grade, and molecular subtype. Patients with advanced disease had significantly higher expression of the HGF and ANGPT2 genes compared to patients with early-stage carcinoma (Fig 2B and 2F). Patients with low-to-moderate grade carcinoma had significantly higher expression of *FGF1* and *FGF2* compared to their counterparts with high-grade tumors (Fig 2K and 2L). Alternatively, *ANGPT1* and *PDGFB* mRNA levels were significantly higher in patients harboring high-grade compared with low-grade tumors (Fig 2M and 2P). The mean mRNA levels of *FGF2*, *ANGPT1*, *ANGPT2*, and *PDGFA* were significantly elevated in cases diagnosed with non-luminal tumors compared to those with the luminal subtypes (p<0.001, Fig 2). No significant differences in *VEGFA* expression were found based on tumor stage, grade, or molecular subtype.

### Expression of pro-angiogenic genes and overall survival of patients with breast cancer

**Fig 3:**
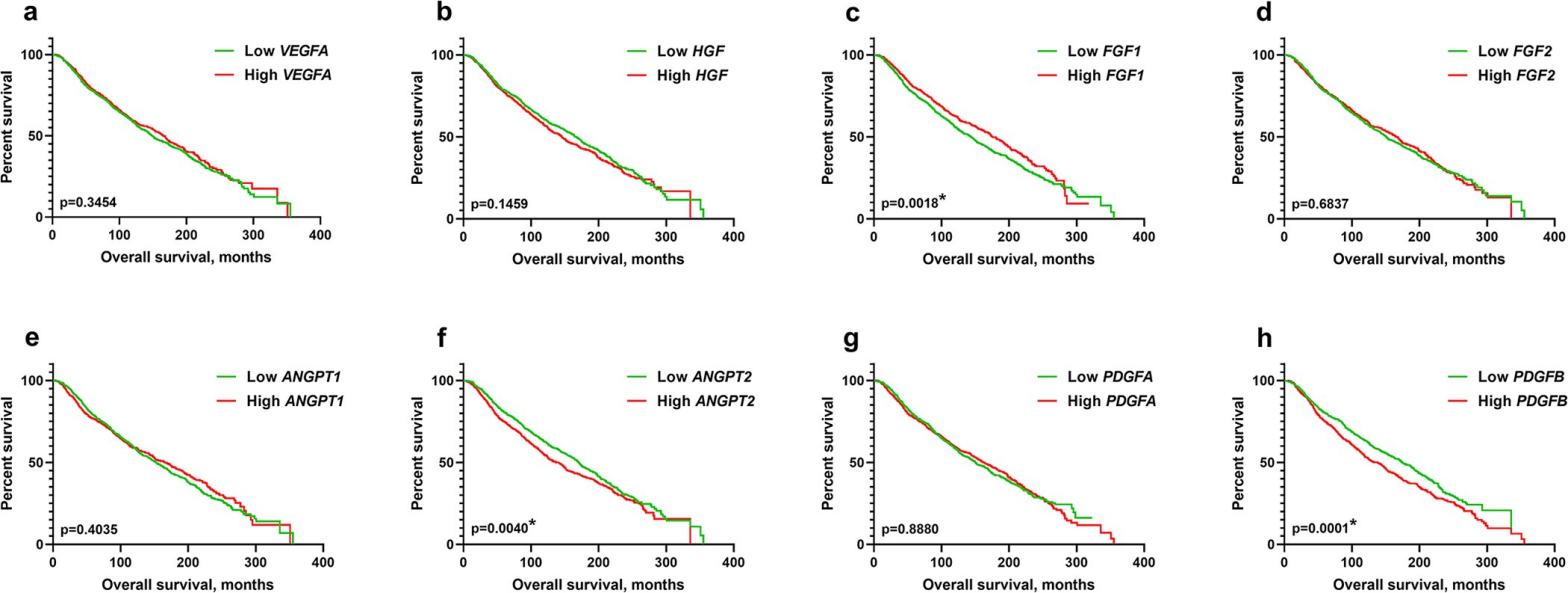
The survival rate according to pro-angiogenic gene expression in patients with breast cancer. Kaplan-Meier survival analyses for breast cancer patients based on the expression status of (a) VEGFA, (b) HGF, (c) FGF1, (d) FGF2, (e) ANGPT1, (f) ANGPT2, (g) PDGFA, and (h) PDGFB genes. *Indicates statistical significance. ANGPT1, angiopoietin 1; ANGPT2, angiopoietin 2; FGF1, fibroblast growth factor 1; FGF2, fibroblast growth factor 2; HGF, hepatocyte growth factor; PDGFA, platelet-derived growth factor A; PDGFB, platelet-derived growth factor B; VEGFA, vascular endothelial growth factor A.

The Kaplan-Meier survival analyses revealed that the OS of patients was significantly affected by the expression status of the genes *FGF1*, *ANGPT2*, and *PDGFB* (p<0.01, Fig 3C, 3F and 3H). High *FGF1* expression correlated with longer median survival time compared to low gene expression (median survival 175.9 and 143.1 months, respectively). In contrast, the *ANGPT2* and *PDGFB* high-expression groups had significantly shorter OS (median survival 140.6 and 137.1 months, respectively) compared to their low-expression counterparts who had significantly longer OS (median survival 170.6 and 173.9 months, respectively). There were no significant differences in OS between low and high-expressing groups of *VEGFA*, *HGF*, *FGF2*, *ANGPT1*, and *PDGFA* among patients with breast cancer. S1 Table summarizes the findings of the Kaplan-Meier survival analysis.

## Discussion

Angiogenesis is a hallmark of cancer. The introduction of angiogenesis inhibitors changed the landscape for the treatment of several types of advanced solid cancers. Nevertheless, the inhibitors of the VEGF/VEGFR pathway failed to improve the outcomes in patients with metastatic breast cancer and the withdrawal of bevacizumab approval for such indication has been a remarkable example [4]. None of the clinically available angiogenesis inhibitors are currently approved for breast cancer. In this study, we investigated the gene expression patterns of 8 different pro-angiogenic genes belonging to 5 different families and their correlation with clinicopathologic characteristics and OS in patients with breast cancer using the METABRIC genomic dataset.

VEGFA is the prototypical and the most characterized member of the VEGF family [37, 38]. Alternative splicing of the *Vegf*a gene pre-mRNA produces distinct VEGFA isoforms, of which 16 have been identified including VEGF_111_, VEGF_121_, VEGF_145_, VEGF_165_, VEGF_189_, and VEGF_206_ [38]. In this analysis, VEGFA was the most abundant pro-angiogenic gene expressed in breast cancer tissues as demonstrated by the mRNA transcript levels. This finding is consistent with previous studies reporting high levels of VEGF expression in breast cancer [11, 27, 28]. Relf et al. showed that VEGF was the most abundant growth factor expressed in breast cancer tissues and that high VEGF levels were associated with poorer survival [39]. Despite the abundant expression of *VEGFA* in breast cancer tissues in our study, it lacked correlation with tumor clinicopathologic characteristics and OS. In a previous analysis of the RNA sequencing data from The Cancer Genome Atlas (TCGA), Ramanathan et al. indicated that *VEGFA* expression was not significantly associated with disease-free survival and OS in patients with breast cancer [40]. These findings are particularly interesting as they give preliminary insights into the lack of efficacy for inhibitors of the VEGF/VEGFR axis in the treatment of breast cancer. It is worth mentioning that the activity of a particular growth factor is fundamentally dependent on the tissue type, the expression level of target receptors, and the disease state [38]. Therefore, the understanding of the biological effect of the different VEGFA isoforms and their role in breast cancer angiogenesis would be critical to developing novel inhibitors of the VEGF/VEGFR pathway [38].

The hepatocyte growth factor (HGF) is a plasminogen-related growth factor secreted by stromal cells. HGF is a well-characterized angiogenic factor stimulating tumor vascularity by promoting the motility and branching morphogenesis of endothelial cells [41, 42]. The biological activity of HGF is mediated by the RTK, MET. Both the ligand and its receptor are upregulated in several tumors including breast cancer and are associated with invasive tumor growth [41, 42]. In this study, *HGF* was the least expressed in breast cancer tissues. The mRNA levels of *HGF* were associated with advanced tumor stage, a known adverse prognosticator. Nevertheless, *HGF* mRNA levels were not correlated with the age of the patient, tumor size, grade, lymph node metastasis, receptor status, and molecular subtype in this analysis. Similar to our findings, Yang et al. indicated that HGF expression in breast cancer tissues was not associated with patient age, size of tumor, or hormone receptor status, rather, its expression was associated with the TNM clinical stage, grade, and lymph node metastasis [43]. Recent reports revealed that the prognostic impact of HGF in breast cancer could be determined by the race of patients. Jones et al. showed that HGF expression was a defining feature of basal-like tumors, and it was associated with the black race and young women [44]. Evidence from previous studies showed that HGF-induced VEGF expression in different cancer cells [42, 45]. Zhang et al. reported increased levels of VEGF after exposure to HGF in the triple-negative MDA-MB-231 breast cancer cell line in vitro [46]. In our study, however, the expression levels of *VEGFA* and *HGF* were not correlated. Rather, *HGF* mRNA levels correlated negatively with the levels of both *ANGPT2* and *PDGFA*.

The mammalian fibroblast growth factor (FGF) family consists of 23 related polypeptides that act by binding to their target RTK family [47]. FGF1 and FGF2, originally called acidic and basic FGFs, respectively, were the first to be discovered and characterized. FGFs are key regulators of cell migration and angiogenesis [47]. FGF1 is an endothelial cell growth factor and a known inducer of angiogenesis [48]. FGF2 angiogenic activity is mediated by inhibiting apoptosis of endothelial cells and promoting their proliferation and migration [47]. In this study, the mRNA expression levels of *FGF1* and *FGF2* were comparable, however they were not correlated. Interestingly, the expression pattern of the FGFs was oppositely correlated with the age of the patient, in which *FGF1* and *FGF2* were correlated negatively and positively with the age at diagnosis, respectively. Thus, it can be concluded that the contribution of FGF1 to breast cancer angiogenesis is dominant at a young age compared to FGF2 which plays a greater role at an older age. Besides, the impact on tumor clinicopathologic characteristics was variable. Our findings indicated favorable associations for *FGF1* expression with reduced tumor size, lower grade, hormone receptor positivity, and increased OS. Alternatively, *FGF2* expression was associated with better prognosis in terms of reduced NPI, lower tumor grade, and HER2-negative status. In contrast to our findings, FGF1 expression has been shown to induce tumor growth and metastasis in previous studies [39, 49, 50]. Okunieff et al. showed that overexpression of FGF1 in breast cancer cells was associated with increased metastasis to the lungs [51].

The angiopoietin (ANGPT) family is an important group of vascular endothelial growth factors, whose functions are mediated through two RTKs, Tie1 and Tie2 [52, 53]. ANGPT1 is essential for vascular integrity, whereas ANGPT2 plays a role in vascular leakage and instability [52, 53]. Our results indicated high expression of both ANGPT genes which were positively correlated in terms of gene expression. In agreement, Rykala et al. reported extremely high levels of ANGPT2 in breast tumors [54]. mRNA expression of *ANGPT1* correlated positively with the expression of *FGF1* and *FGF2* in our study. Likewise, Hayes et al. indicated that ANGPT1 and FGF1 were positively co-expressed in breast cancer cell lines [55]. *ANGPT1* expression correlated negatively with the age of patients at diagnosis suggesting higher mRNA levels among younger patients. Further, *ANGPT1* expression correlated positively with NPI implying poor prognosis among patients in our study. Expression of both genes was also associated with hormone receptor negativity and the non-luminal subtypes. In agreement with our findings, earlier studies revealed a distinct prognostic impact for ANGPTs in breast cancer depending on molecular subtypes and other clinical factors [40, 55, 56]. A clinical sample database showed that ANGPT1 promoted the proliferation of triple-negative breast cancer cells [57]. Additionally, higher levels of ANGPT1 in triple-negative tumors were correlated with poor prognosis compared to other subtypes [57]. A positive association between ANGPT2 expression and lymph node metastasis in breast tumor samples was indicated by Sfiligoi et al. [58]. In their analysis, patients with high *ANGPT2* mRNA expression had shorter OS compared to those with low expression. In concordance with our findings, high ANGPT2 gene expression was associated with reduced disease-free survival and OS in patients with breast cancer [40, 58].

The platelet-derived growth factor (PDGF) family consists of 4 different polypeptide growth factors named A, B, C, and D [59]. Five dimeric compositions for the factors were identified: PDGF-AA, -BB, -AB, -CC, and -DD. The biological activity of the PDGFs is mediated by two distinct RTKs, PDGFRα and PDGFRβ [59]. Activation of the PDGF/PDGFR pathway is associated with cancer cell growth, migration, and angiogenesis [60]. Both *PDGFA* and *PDGFB* were expressed in breast tumor samples in this study and their mRNA transcript levels were positively correlated. High levels of PDGF expression were previously described in breast tumors [54, 61]. The expression of the PDGF genes was differently associated with the clinicopathologic features of breast tumors in our analysis. *PDGFA* expression was inversely correlated with the age of the patient, lymph node metastasis, and the NPI. In contrast, Liu et al. reported that the expression PDGFA was associated with lymph node metastasis in breast cancer [61]. Our findings revealed higher levels of *PDGFB* mRNA among patients with high-grade, progesterone receptor-negative, and HER2-positive tumors. Alternatively, no correlations were found between PDGFB expression and tumor grade, hormone receptor status, and HER2 status in breast tumors in a previous study [62]. While *PDGFA* mRNA levels did not impact survival rates in our analysis, patients with low *PDGFB* mRNA levels had longer OS compared to those with high expression. In line with our findings, a previous study demonstrated that breast cancer patients who had positive tissue immunostaining for PDGF had shorter survival than those with no immunostaining [63]. Clearly, the impact of the expression of the different pro-angiogenic factors on breast cancer clinicopathologic characteristics and prognosis is variable. Such variability could be explained, at least in part, in terms of the different populations studied and the different methodological techniques used to assess the expression levels of the genes and proteins of interest. Nevertheless, findings from literature urge the need to better understand tumor vasculature in breast cancer to delineate the pathways most associated with angiogenic switch and vascular growth.

In light of our results, targeting HGF, ANGPT, and PDGF pathways could provide an effective strategy to inhibit angiogenesis in breast cancer. Previous studies showed that combination therapy using HGF and VEGF inhibitors effectively suppressed tumor angiogenesis [38]. Targeting the HGF/MET axis could be promising for sensitizing tumors to VEGF inhibitors like bevacizumab and overcoming anti-angiogenic treatment resistance [64]. In the same context, targeting ANGPTs as monotherapy or in combination with VEGF/VEGFR inhibitors demonstrated effective anticancer activity in different tumor models [40, 53]. A study on an experimental breast cancer model showed that the combined inhibition of VEGF and ANGPT2 significantly reduced the number of brain metastatic lesions and their permeability [65]. Co-targeting the tyrosine kinase domains of multiple angiogenic factor receptors may achieve favorable outcomes in cancer therapy. This approach could potentially inhibit multiple signaling pathways that promote tumor growth and metastasis. For instance, dovitinib (TKI258), an inhibitor of the receptors of VEGF, FGF, and PDGF, produced antitumor activity and reduced tumor growth in breast cancer models [66, 67]. The strategy of the combination of angiogenesis inhibitors provides the advantage of overcoming the known variability in the patterns and characteristics of tumor vascularity and hence the variability in the response to angiogenic inhibitors [68]. Besides, concomitant targeting of different angiogenesis pathways reduces the risks associated with upregulating compensatory angiogenic signaling pathways associated with acquired resistance to individual inhibitors [2]. Based on our findings, the gene expression of ANGPTs and PDGFs was associated with non-luminal molecular subtypes and hormone receptor negativity. This is particularly interesting as these pathways could be further investigated in patients with triple-negative tumors who lack the classical targets in breast cancer treatment including hormone receptors and HER2 allowing a personalized approach to therapy. Nevertheless, the strategy for combining angiogenesis inhibitors needs extensive investigations to identify the most effective combinations and evaluate their potential toxicity as previous studies indicated that therapeutic angiogenesis inhibitors were associated with increased local invasiveness and distant metastasis of primary tumors [69]. In another avenue, targeting HGF, ANGPTs, and PDGFs could be investigated in combination with other anticancer drugs such as cytotoxic chemotherapy, targeted therapy, and immune therapy. In accordance, various non-VEFG/VEGFR angiogenesis inhibitors are being investigated in patients with breast cancer in clinical settings. In a phase II trial, futibatinib, a potent selective FGF receptor 1–4 inhibitor, is being evaluated as monotherapy and in combination with fulvestrant in patients with advanced or metastatic breast cancer harboring FGF receptor gene aberrations (NCT04024436). Rogaratinib is another potent and selective FGF receptor inhibitor. Its efficacy and tolerability are being investigated in phase II, open-label study in patients who were enrolled in rogaratinib studies (NCT04125693). Trebananib is an ANGPT1/2-neutralizing antibody and the first drug to target the ANGPT/Tie-2 signaling pathway [70]. An open-label, phase II trial evaluating the efficacy of trebananib alone or in combination with standard targeted therapies in patients with breast cancer is currently recruiting patients (NCT01042379). Table 4 summarizes the ongoing clinical trials for selected non-VEGF/VEGFR angiogenic inhibitors in breast cancer.

**Table 4:** Ongoing clinical trials for non-VEGF/VEGFR angiogenesis inhibitors in breast cancer [retrieved from: www.clinicaltrials.gov].

Clinical trial identifier	Phase	Status	Treatment	Objectives
FGFR inhibitors				
NCT03238196	Ib	Active, not recruiting	Fulvestrant, palbociclib, and erdafitinib	Safety, tolerability, and antitumor activity
NCT02052778	I/II	Active, not recruiting	Futibatinib	Safety, tolerability, and antitumor activity
NCT04024436	II	Active, not recruiting	Futibatinib and fulvestrant	Efficacy and safety
NCT03344536	I/II	Completed	Debio 1347 and fulvestrant	Efficacy and dose-limiting toxicity
NCT04483505	I	Completed	Rogaratinib, palbociclib, and fulvestrant	Identify the recommended dose and safety
NCT04125693	II	Completed	Rogaratinib	Safety and tolerability
NCT04526106	I	Recruiting	RLY-4008	Maximum tolerated dose and tolerability
NCT04504331	Ib	Terminated	Infigratinib and tamoxifen, or fulvestrant and palbociclib	Identify dose-limiting toxicity
NCT05560334	II	Recruiting	Pemigatinib	Efficacy and safety
NCT02393248	I/II	Terminated	Pemigatinib and anticancer drugs	Maximum tolerated dose and efficacy
**Angiopoietin inhibitors**				
NCT01042379	II	Recruiting	Trebananib and standard therapies	Efficacy of treatment
NCT00511459	II	Completed	AMG 386, paclitaxel, and bevacizumab	Safety, tolerability, and antitumor activity
NCT00807859	Ib	Completed	AMG 386	Maximum tolerated dose
**PDGFR inhibitors**				
NCT04771520	II	Recruiting	Avapritinib	Safety and efficacy

FGFR, fibroblast growth factor receptor; PDGFR, platelet-derived growth factor receptor.

Our findings add to the growing evidence about the complexity and heterogeneity of angiogenic pathways regulating vascular growth in solid cancers. The remarkable associations between the ANGPT and PDGF genes with the clinical features and outcomes of breast cancer in our study highlight the need to expand the research on tumor angiogenesis by exploring alternate signaling pathways. This is particularly critical in solid cancers lacking a clear therapeutic advantage for the classical VEGF/VEGFR inhibitors, thus delineating new molecular targets in tumor angiogenesis. Besides, our results suggest that the tumoral expression profile of pro-angiogenic genes could serve as a novel prognostic biomarker to point out patients who are at higher risk of poor outcomes and could be considered for more aggressive treatment. To better achieve such goals, preclinical studies in breast cancer models are necessary to understand the role of the different pro-angiogenic signaling pathways, their interactions, and outcomes of targeting.

This study has some limitations. First, our findings are based exclusively on the mRNA expression levels of the selected pro-angiogenic genes in clinical breast cancer samples. Despite the robust remarks that can be withdrawn from gene expression data, the mRNA transcript levels may not fully correlate with the functional protein product which is the ultimate form of the angiogenic growth factor linked to its biological and cellular activities. Therefore, the assessment of the protein levels and activity of these pro-angiogenic growth factors in clinical breast cancer samples is critical to integrate the proteomic findings with the genomic data to provide a deeper understanding of the prognostic role of the different pro-angiogenic factors and their functional interactions in breast cancer. Second, the observational nature of this analysis limits the ability to infer causality between the gene expression patterns and the clinical outcomes. Hence, prospective, multicenter studies are essential to expand our findings across diverse patient populations. Third, our results are restricted to a single time point at the time of diagnosis of breast cancer. Longitudinal studies that monitor changes in expression profiles for the target genes could offer further insights into the temporal and treatment factors that affect the utilization of pro-angiogenic genes as biomarkers and potential drug targets. Accordingly, the next obvious future direction should include further validation of these preliminary findings utilizing large-scale clinical breast tumor samples for both genomic and proteomic analysis of pro-angiogenic growth factors and their relationship with clinicopathologic features and outcomes in patients.

## Conclusion

This study described the expression of 8 pro-angiogenic genes in breast cancer tissues and their association with tumor features and survival using large data from the METABRIC dataset. Our findings indicate no association of *VEGFA* expression with survival and clinicopathologic characteristics in this cohort of breast cancer patients. *FGF1* and *FGF2* expression demonstrated a favorable prognostic impact. Alternatively, expression of *ANGPTs* and *PDGFs* adversely affected survival and prognosticators in patients which together could make these pathways valid targets for the development of novel angiogenesis inhibitors. Collectively, this analysis highlights the complex interactions between the different pro-angiogenic genes and their impact on disease characteristics and prognosis. Further investigations are required to fully understand the signaling pathways associated with angiogenesis in breast cancer to better identify potential therapeutic targets.

## Supporting information

S1 Table Survival analysis findings based on pro-angiogenic gene expression status in patients with breast cancer. (DOCX)
