# Supplementary material for: Exploring angiogenic pathways in breast cancer: Clinicopathologic correlations and prognostic implications based on gene expression profiles from a large-scale genomic dataset
Source: PLoS One. 2024 Sep 20;19(9):e0310557. doi: 10.1371/journal.pone.0310557 (PMC11414925; doi:10.1371/journal.pone.0310557)
Supplement: S1 Table — (DOCX) [file pone.0310557.s001.docx]

**S1 Table. Survival analysis findings based on pro-angiogenic gene expression status in patients with breast cancer.**

| Gene expression status | Median survival (months) | 95% CI | p value |
| --- | --- | --- | --- |
| *VEFGA* Low | 148.6 | 0.7977 to 1.014 | 0.3454 |
| *VEGFA* High | 165.2 |  |  |
| *HGF* Low | 165.4 | 1.011 to 1.280 | 0.1459 |
| *HGF* High | 145.4 |  |  |
| *FGF1* Low | 143.1 | 0.7212 to 0.9181 | 0.0018* |
| *FGF1* High | 175.9 |  |  |
| *FGF2* Low | 150.7 | 0.7982 to 1.019 | 0.6837 |
| *FGF2* High | 167.1 |  |  |
| *ANGPT1* Low | 152.3 | 0.8154 to 1.043 | 0.4035 |
| *ANGPT1* High | 165.2 |  |  |
| *ANGPT2* Low | 170.6 | 1.079 to 1.366 | 0.0040* |
| *ANGPT2* High | 140.6 |  |  |
| *PDGFA* Low | 150.6 | 0.8260 to 1.047 | 0.8880 |
| *PDGFA* High | 161.9 |  |  |
| *PDGFB* Low | 173.9 | 1.127 to 1.427 | 0.0001* |
| *PDGFB* High | 137.1 |  |  |

ANGPT1, angiopoietin 1; ANGPT2, angiopoietin 2; FGF1, fibroblast growth factor 1; FGF2, fibroblast growth factor 2; HGF, hepatocyte growth factor; PDGFA, platelet-derived growth factor A; PDGFB, platelet-derived growth factor B; VEGFA, vascular endothelial growth factor A.

*Indicates statistical significance.
